# Does human bocavirus infection depend on helper viruses? A challenging case report

**DOI:** 10.1186/1743-422X-8-417

**Published:** 2011-08-29

**Authors:** Monika Streiter, Monika Malecki, Aram Prokop, Verena Schildgen, Jessica Lüsebrink, Andreas Guggemos, Matthias Wißkirchen, Michael Weiß, Reinhold Cremer, Michael Brockmann, Oliver Schildgen

**Affiliations:** 1 Klinik für Kinder- und Jugendmedizin, Kinderkrankenhaus, Kliniken der Stadt Köln gGmbH, Amsterdamer Str. 59, Cologne, 50735, Germany; 2 Institut für Pathologie, Krankenhaus Merheim, Kliniken der Stadt Köln gGmbH, Klinikum der Privaten Universität Witten/Herdecke, Ostmerheimer Str.200, Cologne, 51109, Germany

## Abstract

A case of severe diarrhoea associated with synergistic human bocavirus type 1 (HBoV) and human herpes virus type 6 (HHV6) is reported. The case supports the hypotheses that HBoV infection under clinical conditions may depend on helper viruses, or that HBoV replicates by a mechanism that is atypical for parvoviruses, or that HBoV infection can be specifically treated with cidofovir.

## Background

The human bocavirus (HBoV) was discovered in 2005 by the Swedish group of Tobias Allander and colleagues [1]. By now, HBoV was detected in patients suffering from respiratory infections and gastrointestinal diseases, but a proof that HBoV is the causative agent in such cases is missing as it remains impossible so far to fulfil Koch's modified postulates [2]. The latter problem is caused by the fact that HBoV is hardly to propagate in cell culture [3] and that no transmission within an animal model was successful until today.

Since HBoV infections are accompanied by co-pathogens in a very high frequency it was postulated that HBoV is a passenger rather than a pathogen in airway infections [2], despite of the fact that HBoV causes a productive infection with viral shedding, viremia, and putatively persistence in different organs [4-7]. Although HBoV meanwhile was classified as an autonomous parvovirus [3] rather than a Dependovirus like the Adeno-associated virus (AAV) there remains the possibility that HBoV infections depend on helper viruses or at least contributes synergistically to the clinical course. Dependoviruses require a helper virus such as the polyomavirus SV40, herpes viruses, or adenoviruses, of which SV40 and Herpesviruses are known to be able to initiate a rolling circle genome replication [8-12]. A recent study has demonstrated that HBoV in turn is capable to form head-to-tail DNA-sequences that on the other hand are a typical feature of a rolling circle replication. The presented case report provides evidence that the HBoV infection is probably dependent on herpesvirus replication or sensitive to anti-herpesviral therapy with the nucleotide analogue cidofovir.

## Case Report

The patient is a 14 months old Caucasian male with a known history of a myelosarcoma with affected bone marrow, recurrent HHV6 pneumonia, and multiple hospitalisations for several reason. Of note, the patient also suffers from a secondary immunodeficiency with predominant global lack of Immunoglobulins A, G, and M, which origin is not yet determined.

In January 2011 the patient was hospitalized for severe pneumonia accompanied by Norovirus-induced gastroenteritis with severe diarrhoea. Thereby the hospitalisation was required as the symptoms of diarrhoea were recurring since November 2010 and resulted in a stop of weight gain with the risk of dehydration. The patient was released from the hospital 3 days later but was re-hospitalized after four weeks due to aggravation of the gastrointestinal disease.

Despite reduced body weight the patient's general condition was classified as good, however accompanied by moderate anaemic but partially irritated flaked skin, reduced turgor, and wheezing, with radiological signs of an atypical pneumonia (banded shadows in the right upper lope and moderately reduced ventilation). Antibody levels and the blood lymphocyte count were extremely low, whilst monocytes and eosinophiles were significantly increased. Serologically, the patient was tested negative for CMV, EBV, parvovirus B19, and adenoviruses but positive for HHV6 with a viremia increasing from 5.3 × 10^6 ^to 7.8 × 10^6 ^copies per ml serum. Stool samples were tested negative for rotavirus, adenoviruses, norovirus, Clostridium difficile (strains and toxins), Salmonella, Shigella, Yersinia, and Campylobacter coli/jejuni as well as enteropathogenic E. coli.

Due to the ongoing and severe diarrhoea further analyses and pathological investigations were initiated. Thereby, the human herpes virus 6 (HHV6) infection was confirmed in blood, duodenum, and colon biopsies. Instead, human bocavirus was detected in stool and, surprisingly, in duodenum biopsies but not in blood or colon biopsies. Sequencing analyses and subsequent blastN revealed that HBoV1 was present with an E-value of 5 × 10^-68 ^to 3 × 10^-65 ^and a total matching score ranging between 292 and 297. No further signs for a pathological disorder were detected. Based on the pathological and virological diagnoses antiviral therapy was started with Cidofovir. During this therapy, the HHV6 viremia decreased but remained detectable in both serum and stool samples taken in two intervals of 14 days; thereby, most surprisingly, during the Cidofovir therapy HBoV became undetectable in the stool samples 2 weeks after the onset of the antiviral therapy while HHV6 remained positive.

## Discussion

In the recent past human bocavirus has been frequently associated with severe diarrhoea and turned out to be a most frequent agent in this clinical disorder [13-19], leading to the assumption that HBoV is the causative agent despite the missing proof of Koch's postulates for HBoV [2]. Thereby, mainly the genotypes 2-4 but not genotype 1 cause gastrointestinal infections, whilst the latter is mainly found in respiratory secretions. This latter observation is compatible with the fact that a mild respiratory infection with the clinical picture of a beginning atypical pneumonia was observed that was characterized by slight wheezing and radiological signs. The severe diarrhoea may have been a result of a synergistic interaction between the HHV6 infection and the superinfection by human bocavirus. Human herpes virus 6 was described to be a causative agent for prolonged diarrhoea in high risk patients [20]; the HHV6 associated diarrhoea may have been aggravated by the symptoms that are caused by human bocavirus. Although HBoV genotype 1 is mainly associated with respiratory infections it appears possible that in case of immuno-incompetent patients gastro-intestinal symptoms occur that are usually associated with the HBoV genotypes 2-4.

The case described here is interesting and surprising for two additional reasons: First, the presented case gives raise to the hypothesis that HBoV in fact replicates in the gastrointestinal tract, in particular in the duodenum as confirmed by positive PCR results from the clinical material of duodenum but a negative PCR for the colon samples. Thus, it appears likely that a locus for replication of human bocavirus was identified. Second, the observation that HBoV persisted until the onset of Cidofovir therapy is of major interest. On the one hand this observation leads to the hypothesis that HBoV itself is sensitive to Cidofovir and in turn we would have a specific drug available that could be administered in severe clinical cases. Cidofovir was described to display antiviral activity against DNA viruses beyond the family of herpesviruses [21,22]. On the other hand this observation could lead to a more groundbreaking hypothesis. Recently we have shown that in clinical samples DNA sequences were detectable that are of head-to-tail structure [23]. Head-to-tail sequences of viral genomes are a typical feature of a rolling circle replication [24] rather than of a rolling hairpin as postulated for other parvoviruses [25-27]. The hypothesis that HBoV may replicate in a classical rolling circle mechanism is supported by the fact that HBoV genomes are predominantly of negative sense polarity and that solely a minority of HBoV strains additionally encapsidates positive stranded progeny DNA [28]. Notably, rolling circle replication can be initiated by polyomaviruses and herpesviruses including HHV6 [29-34], both known to be able to act as helperviruses for the dependoviruses, a subfamily of parvoviruses that is not able to replicate autonomously [29-35]. The fact that in the presented case HHV6 accompanied the HBoV infection may consequently give raise to the hypothesis that HBoV in fact replicates in a rolling circle mechanism in the inner sense and that rolling circle replication of HBoV may be triggered by helperviruses. This assumption is supported by the fact that HBoV in the majority of clinical cases was described to be accompanied by at least one co-pathogen which in turn led to the ongoing discussion that HBoV is an innocent bystander rather than a true pathogen [36-41]. In concert with the observation that HBoV can replicate autonomously in primary cell culture [3] the present case report goes beyond and leads to the conclusion that HBoV infection can occur autonomously but can be triggered in its severity by co-infections that in turn may modify the molecular replication strategy of HBoV. Although some of the hypotheses presented in this report remain a matter of speculation until it becomes possible to culture human bocavirus in a simplified culture system or in an animal model the presented observation are of major importance for the treatment of severe infections associated with HBoV and as thus deserve foremost attention.

### Ethical Statement

Written informed consent was obtained from the parents to publish the clinical case in anonymized form.

## Competing interests

The authors declare that they have no competing interests.

## Funding

This study was supported by an unrestricted research grant from the Else Kröner-Fresenius Stiftung, Bad Homburg, Germany.

## References

[B1] Allander T Tammi MT Eriksson M Bjerkner A Tiveljung-Lindell A Andersson B Cloning of a human parvovirus by molecular screening of respiratory tract samples Proc Natl Acad Sci USA 2005 102 12891 6 10.1073/pnas.0504666102 16118271 PMC1200281

[B2] Schildgen O Muller A Allander T Human bocavirus: passenger or pathogen in acute respiratory tract infections? Clin Microbiol Rev 2008 21 291 304 table of contents 10.1128/CMR.00030-07 18400798 PMC2292574

[B3] Dijkman R Koekkoek SM Molenkamp R Schildgen O van der Hoek L Human bocavirus can be cultured in differentiated human airway epithelial cells J Virol 2009 83 7739 48 10.1128/JVI.00614-09 19474096 PMC2708629

[B4] Lu X Gooding LR Erdman DD Human bocavirus in tonsillar lymphocytes Emerg Infect Dis 2008 14 1332 4 18680679 10.3201/eid1408.080300 PMC2600388

[B5] Kuethe F Lindner J Matschke K Prevalence of parvovirus B19 and human bocavirus DNA in the heart of patients with no evidence of dilated cardiomyopathy or myocarditis Clin Infect Dis 2009 49 1660 6 10.1086/648074 19863443

[B6] Kantola K Hedman L Allander T Serodiagnosis of human bocavirus infection Clin Infect Dis 2008 46 540 6 10.1086/526532 18199037 PMC7107971

[B7] Manning A Willey SJ Bell JE Simmonds P Comparison of tissue distribution, persistence, and molecular epidemiology of parvovirus B19 and novel human parvoviruses PARV4 and human bocavirus J Infect Dis 2007 195 1345 52 10.1086/513280 17397006 PMC7109978

[B8] Gerspach R Matz B Herpes simplex virus-induced "rolling circle" amplification of SV40 DNA sequences in a transformed hamster cell line correlates with tandem integration of the SV40 genome Virology 1989 173 723 7 10.1016/0042-6822(89)90586-2 2556850

[B9] Gerspach R Matz B Herpes simplex virus-directed overreplication of chromosomal DNA physically linked to the simian virus 40 integration site of a transformed hamster cell line Virology 1988 165 282 5 10.1016/0042-6822(88)90684-8 2838966

[B10] Dorsett D Deichaite I Winocour E Circular and linear simian virus 40 DNAs differ in recombination Mol Cell Biol 1985 5 869 80 2985972 10.1128/mcb.5.4.869-880.1985 PMC366792

[B11] Martin RG Setlow VP The initiation of SV40 DNA synthesis is not unique to the replication origin Cell 1980 20 381 91 10.1016/0092-8674(80)90624-8 6248240

[B12] Sebring ED Kelly TJ Thoren MM Salzman NP Structure of replicating simian virus 40 deoxyribonucleic acid molecules J Virol 1971 8 478 90 4331651 10.1128/jvi.8.4.478-490.1971 PMC376221

[B13] Kapoor A Simmonds P Slikas E Human bocaviruses are highly diverse, dispersed, recombination prone, and prevalent in enteric infections J Infect Dis 2010 201 1633 43 10.1086/652416 20415538 PMC2902747

[B14] Han TH Kim CH Park SH Kim EJ Chung JY Hwang ES Detection of human bocavirus-2 in children with acute gastroenteritis in South Korea Arch Virol 2009 154 1923 7 10.1007/s00705-009-0533-3 19862470

[B15] von Linstow ML Hogh M Hogh B Clinical and epidemiologic characteristics of human bocavirus in Danish infants: results from a prospective birth cohort study Pediatr Infect Dis J 2008 27 897 902 10.1097/INF.0b013e3181757b16 18756188

[B16] Lau SK Yip CC Que TL Clinical and molecular epidemiology of human bocavirus in respiratory and fecal samples from children in Hong Kong J Infect Dis 2007 196 986 93 10.1086/521310 17763318 PMC7111856

[B17] Lee JL Hung JY Han TH Song MO Hwang ES Detection of human bocavirus in children hospitalized because of acute gastroenteritis J Infect Dis 2007 196 994 97 10.1086/521366 17763319 PMC7111883

[B18] Albuquerque MC Rocha LN Benati FJ Human bocavirus infection in children with gastroenteritis, Brazil Emerg Infect Dis 2007 13 1756 58 18217564 10.3201/eid1311.060671 PMC2878208

[B19] Arthur JL Higgins GD Davidson GP Givney RC Ratcliff RM A novel bocavirus associated with acute gastroenteritis in Australian children PLoS Pathog 2009 5 e1000391 10.1371/journal.ppat.1000391 19381259 PMC2663820

[B20] Breddemann A Läer S Schmidt KG Case report: severe gastrointestinal inflammation and persistent HHV-6B infection in a paediatric cancer patient Herpes 2007 14 41 4 17939902

[B21] Mayer TU Marx A Five molecules we would take to a remote island Chem Biol 2010 17 556 60 10.1016/j.chembiol.2010.06.002 20609405

[B22] De Clercq E In search of a selective therapy of viral infections Antiviral Res 2010 85 19 24 10.1016/j.antiviral.2009.10.005 19852983

[B23] Lüsebrink J Schildgen V Tillmann RL Detection of head-to-tail DNA sequences of human bocavirus in clinical samples PLoS One 2011 in press accepted 30.03.2011 10.1371/journal.pone.0019457 PMC3087758 21573237

[B24] Doermann AH T4 and the rolling circle model of replication Annu Rev Genet 1973 7 325 41 10.1146/annurev.ge.07.120173.001545 4593307

[B25] Cotmore SF Tattersall P An asymmetric nucleotide in the parvoviral 3' hairpin directs segregation of a single active origin of DNA replication Embo J 1994 13 4145 52 8076610 10.1002/j.1460-2075.1994.tb06732.x PMC395337

[B26] Tattersall P Ward DC Rolling hairpin model for replication of parvovirus and linear chromosomal DNA Nature 1976 263 106 9 10.1038/263106a0 967244

[B27] Tattersall P Crawford LV Shatkin AJ Replication of the parvovirus MVM. II. Isolation and characterization of intermediates in the replication of the viral deoxyribonucleic acid J Virol 1973 12 1446 56 4586779 10.1128/jvi.12.6.1446-1456.1973 PMC356787

[B28] Bohmer A Schildgen V Lusebrink J Novel application for isothermal nucleic acid sequence-based amplification (NASBA) J Virol Methods 2009 158 199 201 10.1016/j.jviromet.2009.02.010 19428591

[B29] Alazard-Dany N Nicolas A Ploquin A Definition of herpes simplex virus type 1 helper activities for adeno-associated virus early replication events PLoS Pathog 2009 5 e1000340 10.1371/journal.ppat.1000340 19282980 PMC2650098

[B30] Geoffroy MC Salvetti A Helper functions required for wild type and recombinant adeno-associated virus growth Curr Gene Ther 2005 5 265 71 10.2174/1566523054064977 15975004

[B31] Geoffroy MC Epstein AL Toublanc E Moullier P Salvetti A Herpes simplex virus type 1 ICP0 protein mediates activation of adeno-associated virus type 2 rep gene expression from a latent integrated form J Virol 2004 78 10977 86 10.1128/JVI.78.20.10977-10986.2004 15452218 PMC521801

[B32] Farkas SL Zadori Z Benko M Essbauer S Harrach B Tijssen P A parvovirus isolated from royal python (Python regius) is a member of the genus Dependovirus J Gen Virol 2004 85 555 61 10.1099/vir.0.19616-0 14993638

[B33] Thomson BJ Weindler FW Gray D Schwaab V Heilbronn R Human herpesvirus 6 (HHV-6) is a helper virus for adeno-associated virus type 2 (AAV-2) and the AAV-2 rep gene homologue in HHV-6 can mediate AAV-2 DNA replication and regulate gene expression Virology 1994 204 304 11 10.1006/viro.1994.1535 8091661

[B34] Fisher RE Mayor HD The evolution of defective and autonomous parvoviruses J Theor Biol 1991 149 429 39 10.1016/S0022-5193(05)80091-8 1648152

[B35] Berns KI Parvovirus replication Microbiol Rev 1990 54 316 29 2215424 10.1128/mr.54.3.316-329.1990 PMC372780

[B36] Weissbrich B Neske F Schubert J Frequent detection of bocavirus DNA in German children with respiratory tract infections BMC Infect Dis 2006 6 109 10.1186/1471-2334-6-109 16834781 PMC1550408

[B37] Chow BD Esper FP The human bocaviruses: a review and discussion of their role in infection Clin Lab Med 2009 29 695 713 10.1016/j.cll.2009.07.010 19892229 PMC7125628

[B38] Tan BH Lim EA Seah SG The incidence of human bocavirus infection among children admitted to hospital in Singapore J Med Virol 2009 81 82 9 10.1002/jmv.21361 19031441 PMC7166333

[B39] Zhang LL Tang LY Xie ZD Human bocavirus in children suffering from acute lower respiratory tract infection in Beijing Children's Hospital Chin Med J (Engl) 2008 121 1607 10 19024084

[B40] Garcia ML Calvo C Pozo F Perez-Brena P Vazquez MC Casas I Detection of human bocavirus in ill and healthy Spanish children: a 2-year study Arch Dis Child 2009

[B41] Bonzel L Tenenbaum T Schroten H Schildgen O Schweitzer-Krantz S Adams O Frequent detection of viral coinfection in children hospitalized with acute respiratory tract infection using a real-time polymerase chain reaction Pediatr Infect Dis J 2008 27 589 94 10.1097/INF.0b013e3181694fb9 18520973

